# Multi-omics analysis of somatic mutants reveals TCP7 allelically regulates multiple carotenogenic genes in citrus

**DOI:** 10.1186/s43897-025-00193-9

**Published:** 2026-02-10

**Authors:** Xia Wang, Jialing Fu, Lizhi Song, Qingjiang Wu, Qiuying Fang, Yaqing Zhang, Xiuxin Deng, Qiang Xu

**Affiliations:** 1 National Key Laboratory for Germplasm Innovation and Utilization of Horticultural Crops, Huazhong Agricultural University, Wuhan, 430070 People’s Republic of China; 2 Hubei Hongshan Laboratory, Wuhan, 430070 China

**Keywords:** Somatic mutation, Citrus, Carotenoid, Meta-analysis, Chromatin openness, TCP

## Abstract

Somatic mutations are widespread in all organisms and cause abundant phenotypic changes, constituting an important source of variation especially for clonally propagated plants. Somatic mutants can serve as a mutation-introducing method for exploring the regulatory mechanism of mutated trait formation in clonally propagated plants. Red-fleshed and orange-fleshed pomelo varieties derive from independent mutations of the wild-type white-fleshed Guanxi honey pomelo (*Citrus grandis*), offering an ideal system for dissecting core regulatory transcription factors behind the fruit color trait. Here, we performed a meta-analysis comprising genome, transcriptome, DNA methylome and chromatin accessibility assays of the two color mutants and their wild type, resulting in haplotype-scaled pan-genome. The chromatin openness analysis suggested that binding sites for TEOSINTE BRANCHED 1/CYCLOIDEA/PCF (TCP) transcription factors are significantly enriched in the differentially open regions, with different footprint patterns in the mutants and wild type. We identified CgTCP3, CgTCP7, and CgTCP20 as regulators of key carotenogenic genes including *ZDS*, *BCH*, and *NCED2*. Furthermore, *CgTCP7* underwent positive selection in pomelo germplasm, with various alleles differentially affecting carotenogenic gene expression and modulating carotenoid accumulation. We therefore identified the core transcription factor genes responsible for color trait formation, providing resources for breeding varieties with diversified phenotypes.

**Supplementary Information:**

The online version contains supplementary material available at 10.1186/s43897-025-00193-9.

## Core

A meta-analysis of the genome, transcriptome, DNA methylome and chromatin accessibility of wild-type pomelo and two somatic mutants with different fruit flesh color revealed three core TCP transcription factors affected the content of carotenoids by regulating the expression of multiple key carotenogenic genes. GWAS of pomelo populations for fruit carotenoid composition and an evolutionary analysis of citrus germplasms suggested that allelic variation at *TCP7* affects the accumulation of carotenoids in citrus fruits, providing resources for breeding varieties with diversified phenotypes.

## Gene and accession numbers

Sequence data from this article can be found in the database of the citrus genome database (http://citrus.hzau.edu.cn/download.php.) under the accession numbers: *CgTCP3*, *CgTCP7*, *CgTCP20*, *ZDS*, *BCH*, and *NCED2.*

## Introduction

Somatic mutations are spontaneous mutations that occur in somatic cells rather than germ cells. Somatic mutations can be single-nucleotide polymorphisms (SNPs), insertions and deletions (InDels), or structural variations such as copy number variations (CNVs) and translocations, or can alter epigenetic modifications (Foster and Aranzana 2018). Somatic mutations can be induced by various factors, including DNA replication errors, environmental stress and DNA damage responses, and are a significant source of phenotypic diversity within an organism (Foster and Aranzana 2018).

For asexually reproducing crops, somatic mutations are an important source of variation arising during bud formation, and fruit trees such as citrus are ideal species for exploring the regulatory mechanism of mutated trait formation (Wang et al. 2021). Indeed, in the past 60 years, 88.5% of citrus varieties have originated from bud mutation breeding in China, resulting in a diverse group of varieties with rich trait variations, such as sweet oranges (*Citrus sinensis*), clementine mandarins (*Citrus clementina*), grapefruits (*Citrus paradisi*) and Satsuma mandarins (*Citrus unshiu*) (Deng 2022). The abundant variation resources in citrus are in need of exploration to guide genetic improvement practices and identification of the key genes regulating the formation of mutated traits. For example, the high accumulation of anthocyanins in blood orange is due to an insertion of a *Copia*-like retrotransposon into the promoter of the *Ruby* gene, encoding a MYB-type transcription factor activating the expression of anthocyanin biosynthetic genes (Butelli et al. 2012). The past two decades have, however, seen only a few reports analyzing the characteristics of citrus somatic mutations, most focusing on changes in the expression patterns of known functional genes (Alós et al. 2008; Liu et al. 2007a, b; Rios et al. 2010). For instance, downregulation of the *GARP and coiled-coil 1* (*CcGCC1*) gene, encoding another MYB-type transcription factor, was reported in a clementine mutant with a delayed green-to-orange color shift through transcriptome analysis (Rios et al. 2010).

Based on pairwise comparisons between somatic mutants and wild-types with nearly identical genetic background, somatic mutants can serve as an alternative mutation-introducing method for identifying regulatory genes of mutated traits. A meta-analysis using multi-omics approaches is an optimal strategy for detecting regulatory genes and their mechanisms related to mutated traits (Vandereyken et al. 2023). Multi-omics strategies comprehensively measure changes in different biomolecules, such as metabolites, proteins, RNA, and DNA; explore their interactions and relationships; and can reveal the key regulatory mechanisms in somatic mutants. Recent advances in omics technologies, including sequencing and analysis based on deep learning, have allowed for an unprecedented exploration of the somatic variation landscape. There are two powerful tools for the meta-analysis of somatic mutations: sequencing of highly accurate long reads (HiFi), which significantly improves the accuracy of DNA mutation detection (Rabanal et al. 2022; Yang et al. 2019; Zou et al. 2022), and the assay for transposase-accessible chromatin with sequencing (ATAC-seq), for the identification of key transcription factors causing phenotypic differences (Grandi et al. 2022; Luo et al. 2022; Yan et al. 2020).

Guanxi honey pomelo (*Citrus grandis*), originating from the Fujian Province of China, is a well-known Chinese geographical indication protection product. It boasts a series of excellent bud mutation varieties with superior flavor quality and attractive color appearance (Liu et al. 2019), including varieties with red or orange flesh, red or orange outer peel, and both red flesh and peel. These naturally occurring somatic mutants serve as a treasure trove for understanding the molecular mechanisms underlying color diversity. Studying the composition and contents of carotenoids in fruits from these varieties is of particular interest due to their influence on consumer preference, marketability and fruit nutritional value. A combination of biochemical, molecular biology, genetics and genomics approaches, have led to the identification of the genes related to the main pathways of carotenoid metabolism and elucidation of the underlying biosynthetic pathways (Sun et al. 2022). As a branch of the isoprenoid metabolic pathway in plants, carotenoid metabolism is affected by plant species, developmental stage and various external environmental cues (Sun et al. 2017). Key genes in carotenoid metabolism are regulated at the transcriptional and post-transcriptional levels (Nisar et al. 2015; Yuan et al. 2015). Carotenoid metabolism can also be influenced by plastid differentiation and changes in storage structure of carotenoids, such as preventing plastid division in *orange* (*or*) mutants (Li et al. 2001, 2012). Regulatory factors are themselves regulated, such as photoreceptors in the light signal transduction pathway, transcription factors regulating fruit development, sugars as signaling molecules and genes that regulate the carotenoid pathway post-transcriptionally (Rao et al. 2024).

Taking wild-type Guanxi honey pomelo and two of its somatic mutants with red or orange flesh as materials, we aimed to reveal the genomic and epigenetic characteristics of somatic mutation, and identify the transcriptional regulatory mechanism of somatic mutated phnotype through multi-omic analysis.

## Results

### Red- and orange-fleshed mutants arose from independent somatic mutation events in a wild-type pomelo

**Fig. 1:**
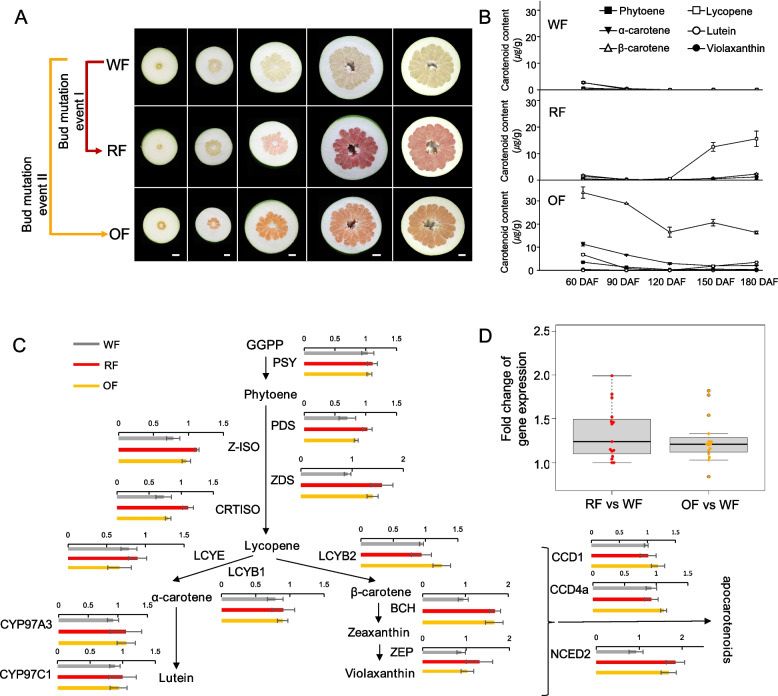
Changes in pigment contents and expression of carotenogenic genes in Guanxi pomelo fruit color mutants. **A** Representative photographs of pomelo fruits from the white-fleshed wild type and the two flesh color mutants of Guanxi pomelo during fruit development (at 60, 90, 120, 150, and 180 DAF). Scale bar, 2 cm. **B** Carotenoid composition and content during fruit development of wild-type Guanxi pomelo and the two color mutants. **C** Relative gene expression along carotenoid biosynthetic pathway in red-fleshed and orange-fleshed pomelo fruits compared to white-fleshed pomelo fruits based on RT-qPCR during the color transition period. Values are means ± SD (*n* = 3). **D** Boxplots of the fold change in gene expression of carotenogenic genes in red-fleshed and orange-fleshed pomelo fruits compared to white-fleshed pomelo fruits based on RT-qPCR. Each dot represents a carotenoid biosynthesis gene. DAF, days after flowering; RF, red-fleshed; OF, orange-fleshed; WF, white-fleshed

Red-fleshed and orange-fleshed Guanxi honey pomelo were selected from wild white-fleshed Guanxi honey pomelo in 2006 and 2013, respectively, suggesting that the latter experienced at least two independent somatic mutation events, both leading to altered flesh color (Fig. 1A). We determined the contents of the main carotenoids—lycopene, α-carotene, β-carotene, lutein and violaxanthin—in these three materials. In the wild type (white-fleshed Guanxi honey pomelo), the total carotenoid content was extremely low throughout all developmental stages tested. The lycopene content in the red-fleshed mutant increased dramatically starting at 120 days after fertilization (DAF) and remained high until maturity. The β-carotene content of the orange-fleshed mutant was high as early as the young fruit stage (60 DAF) (Fig. 1B). Mature red-fleshed fruits accumulated abundant lycopene in the juice sacs, whereas orange-fleshed fruits accumulated high levels of β-carotene, likely explaining their difference in flesh color.

We assessed the expression levels of carotenoid biosynthetic genes in the wild type and its two color mutants during the critical color transition period by reverse-transcription quantitative PCR analysis (RT-qPCR). Among carotenoid biosynthetic genes, *ZETA-CAROTENE DESATURASE* (*ZDS*), *BETA CAROTENOID HYDROXYLASE* (*BCH*) and *NINE-CIS-EPOXYCAROTENOID DIOXYGENASE 2* (*NCED2*) were significantly upregulated in red-fleshed and orange-fleshed fruits (Fig. 1C and Table S1, fold change > 1.5 and *P* value < 0.05). By quantifying the activity level of the entire carotenoid biosynthetic pathway in pomelos with different flesh colors, we found that the pathway activity score of red-fleshed pomelos increased by 1.40 standard deviation units compared to wild-type white-fleshed pomelos, while orange-fleshed pomelos showed an increase of 0.88 units—both exceeding Cohen’s d threshold of 0.8 for a large effect size. Additionally, the expression of almost all key carotenoid biosynthesis genes were higher in the red and orange flesh mutants relative to the white-fleshed wild type (Fig. 1C), with on average a 34% increase in red-fleshed mutant (*P* value, 6.3E − 04; Fig. 1D) and a 26% increase in the orange-fleshed mutant (*P* value, 1.2E − 03; Fig. 1D), suggestive of a more active carotenoid synthesis pathway at the transcriptional level and a stronger carotenoid metabolic flow in the mutants compared to the wild type.

For *LYCOPENE BETA-CYCLASE E* (*LCYE*) and *LCYB2*, whose encoding enzymes are responsible for the conversion of lycopene to α-carotene and β-carotene, respectively, the expression levels of *LCYE* and *LCYB2* expression were comparable in red-fleshed fruits and the white fruits of the wild type (Fig. 1C). Conversely, *LCYB2* showed a modest upward trend (1.33-fold), while *LCYE* showed slight downward trend (0.84-fold), in orange-fleshed fruits compared to the wild type (Fig. 1C). These results suggested that the different expression patterns of key carotenogenic genes may help explain the differences in the flesh colors of the two mutants relative to their common wild type, as they lead to differential allocation between α-carotene and β-carotene biosynthesis.

### Pan-genome analysis revealed no functional variation of carotenogenic genes for the color mutants

To detect whether there are DNA variations that cause changes in flesh color, we assembled a pan-genome from the wild type and the two somatic mutants by combining genome sequencing data from Illumina-based short reads, HiFi long reads and high-resolution chromosome conformation capture (Hi-C) using an iterative assembly strategy (Table S2). This resulted in a high-quality chromosome-scale pan-genome of Guanxi honey pomelos of 335.3 Mb in size, with a longest contig of 49.7 Mb and a contig N50 value of 32.6 Mb (Table 1). Through de novo annotation, annotation based on homology, and transcriptome analysis, we annotated 24,888 genes and 37,367 transcripts (Table 1). We evaluated the completeness of our genome assembly by calculating the Benchmarking universal single-copy orthologs (BUSCO) score, which was 98.2%. Moreover, we produced a haplotype-resolved pan-genome with two haplotypes (Table 1), with a 98.8% identity between the two haplotype sequences.

**Table 1:** Summary statistics of the assembled pan-genome for color mutants of Guanxi pomelos (GX) and its two haplotypes (GX-Haplotype A and GX-Haplotype B)

	**GX**	**GX-Haplotype A**	**GX-Haplotype B**
Total genome size (bp)	335,270,298	316,409,287	318,562,071
Chromosome size (bp)	311,484,155	309,208,490	314,059,868
Longest contig length (bp)	49,717,726	44,498,214	28,739,905
Contig N50 length (bp)	32,583,218	4,296,306	3,948,203
Contig N90 length (bp)	433,629	94,060	476,321
Complete BUSCO (%)	98.2	98.4	97.9
Percentage of repeat elements (%)	55.2	52.62	52.7
Number of protein-coding genes	24,888	24,730	24,247
Number of annotated mRNAs	37,367	37,304	36,904

We used the long reads produced in this study to identify the genetic variants present in the red-fleshed or orange-fleshed mutants relative to wild type using deep learning technology. We randomly selected 16 SNPs for PCR amplification and validation by Sanger sequencing (Fig. S1). Most of the genetic variation between the color mutants and wild-type Guanxi honey pomelo consisted of point mutations, with 22,416 and 16,680 SNPs in the red-fleshed mutant and the orange-fleshed mutant, respectively (Table S3). Prediction of the effects from all genetic variants on genes and their encoding proteins indicated that most polymorphisms occurred in non-coding regions or did not change the protein sequence (Fig. S2). For the key genes in carotenoid biosynthesis, we observed no changes in sequences of the gene and its upstream 2kb region that might cause structural or functional changes, ruling out mutations in these genes as the mechanistic basis for the flesh color in the two mutants (Fig. S3). These suggested that the color mutants are likely caused by epigenetic variations and differences in transcriptional regulation.

### Transcriptional regulomic profiling revealed the high accumulation of carotenoids is to scavenge excessive oxidizing substances in color mutants

**Fig. 2:**
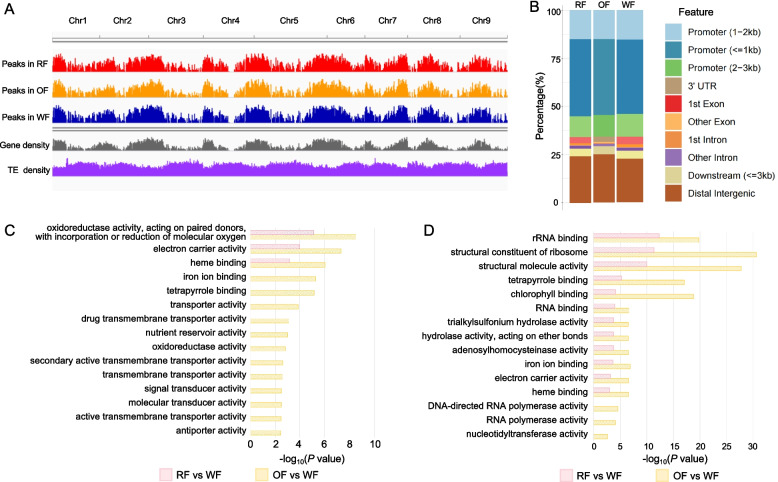
Transcriptional regulatory landscapes among pomelos with different flesh color. **A** Distribution of open chromatin regions across the genome of the wild type and the two color mutants. **B** Percentage of open chromatin peaks in different genomic features in pomelos with different flesh color. **C** Gene ontology (GO) term enrichment analysis of significantly upregulated genes in red-fleshed and orange-fleshed mutants compared to the white-fleshed wild type for molecular functions. **D** GO term enrichment analysis of genes located downstream of differentially accessible chromatin regions in red-fleshed and orange-fleshed mutants compared to the wild type for molecular functions. RF, red-fleshed; OF, orange-fleshed; WF, white-fleshed

We performed ATAC-seq on the flesh of wild-type Guanxi honey pomelo and its red-fleshed and orange-fleshed mutants during the color transition period to quantify the degree of chromatin accessibility. We determined that at the whole-genome scale, the patterns of chromatin accessibility in the mutants and the wild type are almost the same (Fig. 2A). About 65% of all open chromatin regions were located within 3 kb upstream of a transcription start site (TSS), with the region within 1 kb upstream of a TSS showing the highest degree and frequency of chromatin accessibility (Fig. 2B and Fig. S4, accounting for 37%). These results suggest that the region 1 kb upstream of TSSs is the most likely promoter region in pomelo. In addition, chromatin opening in regions beyond 3 kb upstream of a TSS or downstream of a transcription termination site (TTS) was also common (accounting for 24%), suggesting that these regions may contain distal enhancers (Fig. 2B).

Based on our RNA-seq analysis, we identified genes that were differentially expressed in either mutant relative to the wild type. A gene ontology (GO) term enrichment analysis revealed that the genes significantly upregulated in both the red-fleshed and the orange-fleshed mutant are significantly enriched in categories related to oxidoreductase activity, electron carrier activity and heme binding (Fig. 2C). For the orange-fleshed mutant, the upregulated genes were also enriched in categories related to transporter activity and nutritional storage activity (Fig. 2C). We performed a similar enrichment analysis on the genes located downstream of the differentially open chromatin regions identified in the two mutants relative to the wild type. The genes with differential chromatin accessibility in the mutants were significantly enriched in molecular functions related to photosynthesis, electron transport chain and translation (Fig. 2D). Notably, we observed the overlapped enrichment of genes related to the categories ‘electron carrier activity’ and ‘heme binding’ at both the transcriptional level (based on the RNA-seq data) and the transcriptional regulatory level (based on ATAC-seq data). Together with the greater accumulation of carotenoids seen in the mutants, these results suggest that the color mutants may synthesize and accumulate carotenoids to scavenge excessive oxidizing substances, resulting in their red or orange flesh. We also speculate that the plastid-to-nucleus retrograde signaling involving tetrapyrrole biosynthesis, plastid redox state and ROS may regulate the expression of nuclear genes and adjust carotenoid metabolism (Loudya et al. 2024).

### TCPs are core regulatory transcription factors behind the fruit color trait

**Fig. 3:**
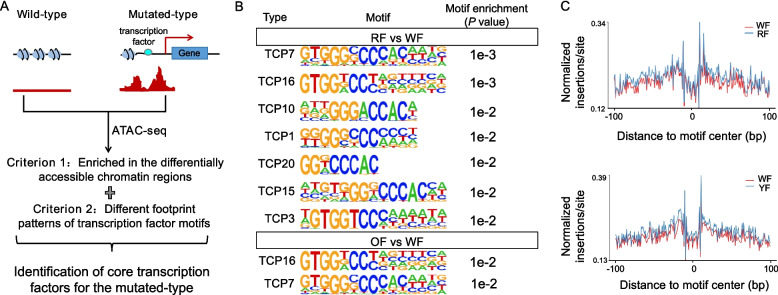
Identification of core regulatory transcription factors associated with fruit color in the mutants of Guanxi pomelo. **A** Principle and method for identifying core regulatory transcription factors associated with fruit color. **B** Enrichment of TCP transcription factor motifs in differentially accessible chromatin regions in red-fleshed and orange-fleshed color mutants compared to the white-fleshed wild type. Enrichment of other transcription factor motifs were provided in Tables S4 and S5. **C** Differential TCP transcription factor footprints in red-fleshed and orange-fleshed color mutants relative to the wild type. The strength of binding signals at the transcription factor binding motif and its upstream and downstream 100-bp regions was measured as the ratio of normalized insertions to total site reads in ATAC-seq data. Transcription factor footprints refer to the protected, low-coverage regions that form when transcription factors bind to specific areas of DNA. Due to the occupancy of space by the transcription factors themselves, they hinder the cutting action of Tn5 transposase in these regions during ATAC-seq experiments, resulting in a ‘notch’ in the data that corresponds to these protected areas. Other transcription factor footprints patterns were provided in Fig. S6 as controls. RF, red-fleshed; OF, orange-fleshed; WF, white-fleshed

To help identify the differences in transcriptional regulation mechanisms in somatic mutants with nearly identical genetic and epigenetic backgrounds, we devised a method using ATAC-seq data to look for core regulatory transcription factors behind the fruit color trait. Such a core regulatory transcription factor should meet two conditions. First, its mutant should result in differences in binding ability to its target genes, detectable as a significant enrichment for its corresponding binding site in the differentially open regions in the mutant as compared to the wild type (Fig. 3A). As the binding of a transcription factor to its downstream targets will block the cleavage of genomic DNA by transposases during the construction of ATAC-seq libraries, a footprint will become detectable for each transcription factor. Thus, such a hypothetical core regulatory transcription factor should exhibit differential binding peaks to its target genes, resulting in distinct footprints between wild type and mutant (Fig. 3A). We therefore performed a combined analysis of enriched transcription factor binding sites and footprints between the mutants and the wild type to identify transcription factors that might be responsible for the different fruit colors of the three genotypes under study (Fig. 3A).

We detected 54 and 44 transcription factors as being enriched within the differentially open chromatin regions identified in red-fleshed and orange-fleshed color mutants relative to the wild type during the color transition period (Fig. 3B and Tables S4 and S5). Of the types of transcription factors identified at this step, only CINCINNATA (CIN)-like TEOSINTE BRANCHED 1/CYCLOIDEA/PCF (TCP) transcription factors exhibited a footprint pattern in the red-fleshed and orange-fleshed mutants that differed from that of the wild type (Fig. 3C), while other types of transcription factor did not show differential footprint patterns (Fig. S5). The above joint analysis suggested that TCP-type transcription factors are likely core regulatory transcription factors affecting flesh color. Looking at the pomelo genomes, we identified genes encoding TCP1, TCP3, TCP7, TCP10, TCP15, TCP16, and TCP20. Based on homology analysis and phylogenetic relationship analysis with *Arabidopsis* TCP gene members, *CgTCP1a* (*Cg9g021490*), *CgTCP1b* (*Cg6g019820*), *CgTCP3* (*Cg4g010590*), and *CgTCP10* (*Cg2g008400*) belong to the Class II subfamily, while *CgTCP7* (*Cg7g014020*), *CgTCP15* (*Cg7g021010*), *CgTCP16* (*Cg8g019820*), and *CgTCP20* (*Cg7g016630*) belong to the Class I subfamily (Fig. S6). After removing all genes with low or no expression in the pulp of wild-type or mutant fruits, we selected four TCP transcription factor genes, namely *CgTCP3*, *CgTCP7*, *CgTCP10* and *CgTCP20*, as candidate core regulatory transcription factors behind the fruit color trait for the somatic mutants of Guanxi pomelo (Table S6).

### TCPs transcriptionally regulate multiple carotenoid biosynthetic genes

The carotenoid biosynthetic genes *ZDS*, *BCH* and *NCED2* were predicted to have TCP-binding sites in their promoters (Table S7), and their expression levels differed among the wild type and the two color mutants. In addition to CgTCP10, other candidate core regulatory transcription factors, namely CgTCP3, CgTCP7 and CgTCP20, were predicted to have binding sites in the promoter of these carotenoid biosynthetic genes (Table S7).

**Fig. 4:**
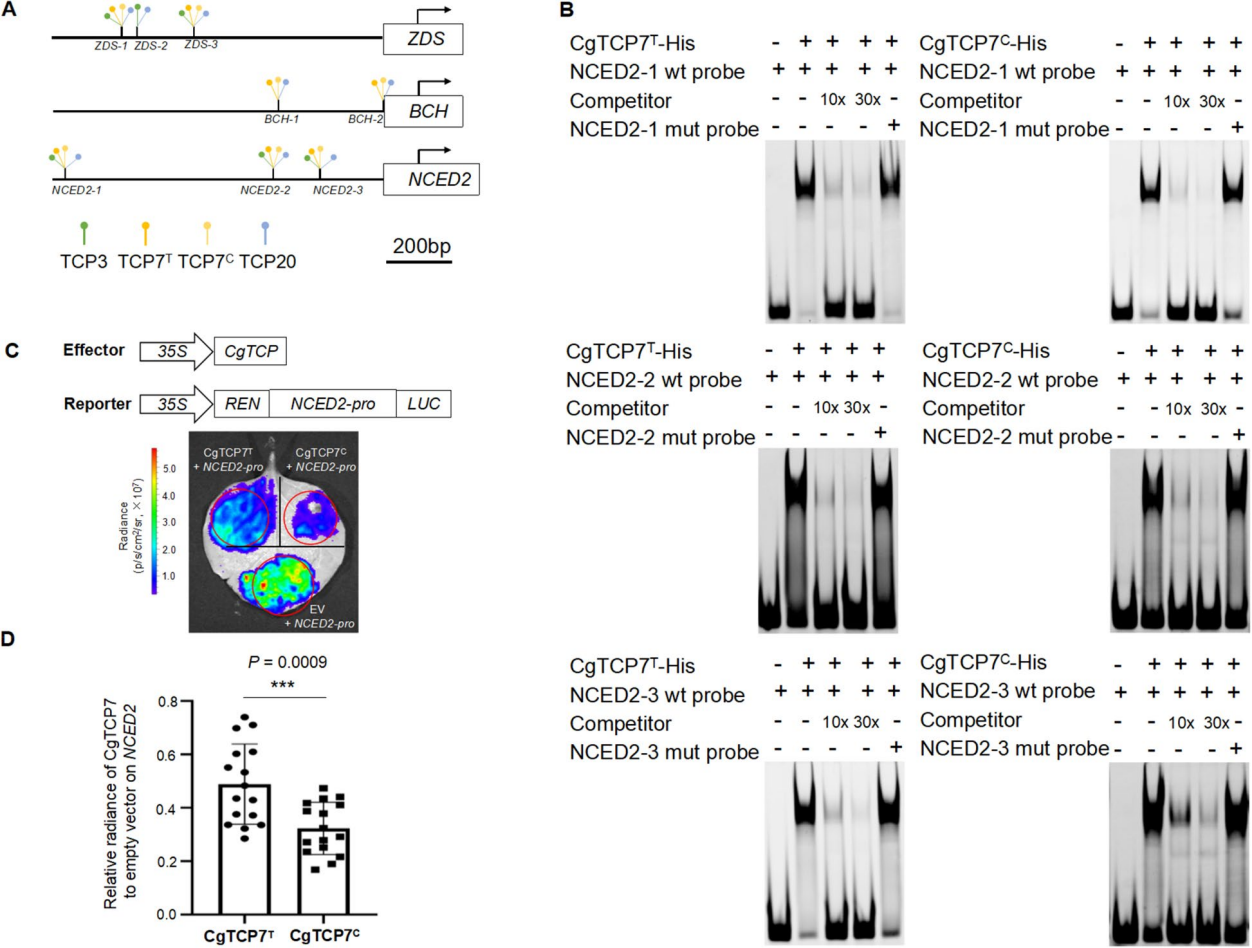
Functional analysis of three core TCPs regulating three carotenoid biosynthetic genes to control carotenoid accumulation. **A** Distribution of TCP-binding sites in the promoters of carotenoid biosynthetic genes generated from electrophoretic mobility shift assay (EMSA). **B** EMSA testing the interaction between recombinant purified CgTCP7 and its target promoter of *NCED2*. **C** Dual-luciferase reporter assay in *N. benthamiana* leaves to test the interaction between CgTCP7 and the *NCED2* promoters. **D** Dual-luciferase assay showing the repression of *NCED2* transcription by CgTCP7^T^ or CgTCP7^C^ in *N. benthamiana* leaves. Values were means ± SD (*n* = 16). The *P* value of significance of difference between CgTCP7^T^ and CgTCP7^C^ based on the t-test was indicated. RF, red-fleshed; OF, orange-fleshed; WF, white-fleshed

To test the binding of each candidate TCP transcription factor to the promoters of these three carotenoid biosynthetic genes, we conducted electrophoretic mobility shift assays (EMSAs) using recombinant purified proteins produced in *E. coli*. In pomelo, *CgTCP7* has two allelic genes, *CgTCP7*^*T*^ and *CgTCP7*^*C*^, with polymorphic bases of T and C respectively in their sequences (Fig. S7). We detected strong physical interactions between CgTCP7^T^, CgTCP7^C^ or CgTCP20 and probes derived from the *BCH* promoters; similarly, we determined that CgTCP3, CgTCP7^T^, CgTCP7^C^ and TCP20 strongly bind to the *ZDS* and *NCED2* promoter (Fig. 4A and B, Fig. S8–10). The *BCH* promoter harbors two TCP-binding sites, while the *ZDS* and *NCED2* promoters each carry three (Fig. 4A). Additionally, the levels of expression found for *CgTCP3*, *CgTCP7*, and *CgTCP20* were compatible with their involvement in pomelo fruit development (Fig. S11). These results suggest that multiple TCPs may be involved in the regulation of carotenoid biosynthetic genes.

Notably, CgTCP7 was the only core transcription factor identified in both the red-fleshed and orange-fleshed mutants based on our chromatin accessibility analysis (Fig. 3B), with two somatic mutation events appearing to have arisen independently. To expand our analysis of the role played by CgTCP7 on transcription, we performed dual-luciferase assays using reporter constructs consisting of the *BCH*, *NCED2* and *ZDS* promoters individually driving the transcription of the firefly luciferase (*LUC*) reporter gene. When we co-infiltrated *Nicotiana benthamiana* leaves with constructs encoding CgTCP7 and each LUC reporter construct, we established that CgTCP7 represses transcription from the *BCH* and *NCED2* promoters, while activating *ZDS* transcription (Fig. 4C and Fig. S12A,B).

### Allelic contribution of positively selected CgTCP7 to carotenoid accumulation in citrus

**Fig. 5:**
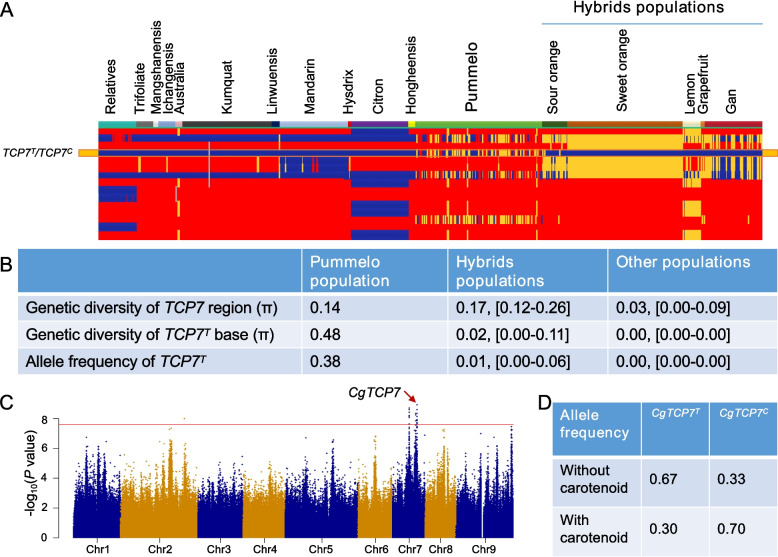
Evolutionary and color-associated analysis of *TCP7* haplotypes/alleles in pomelo and citrus populations. **A** Genotype distribution of the polymorphic sites in the *TCP7* region among 663 accessions from the Citrinae. Each column indicates a citrus accession, and each row indicates a polymorphic base site in the *TCP7* region. Red, yellow and blue represent 0/0, 0/1 and 1/1, respectively. 0 and 1 represent bases that are identical to or different from the reference genome, respectively. Different colors are filled for each column of accessions to represent different citrus categories. **B** Summary table of genetic diversity and allele frequencies at *TCP7* in different citrus populations. **C** Manhattan plot of GWAS for fruit carotenoid composition in the pomelo population. **D** Distribution of *CgTCP7* allele frequency among pomelo accessions with the presence or absence of carotenoids in their flesh

Unlike that of some pomelo accessions, the pulp of most other citrus germplasms is rich in carotenoids and takes on various colors. We collected and analyzed published genome sequences from 663 citrus germplasm resources following a single pipeline to conduct an evolutionary analysis of *CgTCP7* and its orthologs in other citrus germplasms. *TCP7* was highly conserved in different citrus species of the Citrinae, as indicated by the high mappability of reads across this region. The genetic diversity over the *CgTCP7* region in the pomelo population (0.14) was significantly higher than that for *TCP7* in other citrus populations (mean value, 0.03; Fig. 5A, B). Notably, at the position equivalent to the polymorphic nucleotide detected in *CgTCP7*, only pomelo and its descendants (some sour orange accessions) harbored the *TCP7*^*T*^ haplotype, while all other citrus species were homozygous for *TCP7*^*C*^ (Fig. 5A, B). The frequencies of the *TCP7*^*T*^ haplotype in pomelo and in other citrus populations were 0.38 and 0.0, respectively (Fig. 5B). The significant decrease in genetic diversity and skew of allele frequency suggested that *TCP7* has undergone strong positive selection in pomelo, with the *TCP7*^*T*^ haplotype being a pomelo-specific haplotype. Purification selection during the evolution of citrus strongly influenced the haplotype composition at *TCP7* in other citrus populations such as mandarin and lemon, whose fruit flesh is generally rich in carotenoids.

We performed a genome-wide association study (GWAS) for fruit carotenoid composition in the pomelo population, which revealed multiple major genomic regions controlling pomelo flesh color, with the most significant association being two regions mapping to chromosome 7, one on chromosome 9 and one on chromosome 2 (Fig. 5C). Importantly, the physical position of *CgTCP7* overlapped with the significantly associated region on chromosome 7 (Fig. 5C). The *CgTCP7* allele was significantly correlated with the presence or absence of carotenoid in pomelo flesh, with *CgTCP7*^*T*^ occurring in 67% of pomelo accessions without color (Fig. 5D). Furthermore, the dual-luciferase assay indicated that CgTCP7^C^ represses *NCED2* and *BCH* transcription more strongly than CgTCP7^T^ (Fig. 4D and Fig. S12D), while CgTCP7^T^ activates *ZDS* transcription to a greater extent than CgTCP7^C^ (Fig. S12C). We proposed that the differential allelic regulation of carotenogenic genes by the *TCP7* allele probably contributes to the differential coloration of pomelo and other citrus fruits.

## Discussion

We performed a meta-analysis of the genome, transcriptome, DNA methylome and chromatin accessibility of wild-type pomelo and two somatic mutants with different fruit flesh color. Taking these somatic mutants with consistent backgrounds as a system to identify color-related regulatory genes, we identified three core TCP transcription factors that affect the composition and content of carotenoids by regulating the expression of multiple key carotenoid biosynthetic genes. Finally, a GWAS of pomelo populations for fruit carotenoid composition and an evolutionary analysis of citrus germplasms suggested that allelic variation at *TCP7* affects the accumulation of carotenoids in citrus fruits.

The three TCP transcription factors identified in this study were not previously known to affect the expression of carotenoid biosynthetic genes, unlike reported transcription factors regulating carotenoid pathway genes such as MADS3 (Zhu et al. 2023), MADS5 (Lu et al. 2021), MADS6 (Lu et al. 2018), MADS32 (Xia et al. 2023) and DIVARICATA1 (Song et al. 2023). Indeed, the TCPs have mainly been shown to regulate chloroplast biogenesis and thus affect the color of plants in *Arabidopsis* (Sun et al. 2019; Zheng et al. 2022). As to the regulation of secondary metabolites accumulation, TCP3 was reported to interact with R2R3-MYB proteins, thus promoting flavonoid biosynthesis and negatively modulating the auxin response in *Arabidopsis thaliana* (Li and Zachgo 2013), and TCP15 was reported to inhibit anthocyanin accumulation during exposure to high light intensity conditions by modulating the expression of anthocyanin biosynthesis genes in *Arabidopsis* (Viola et al. 2016). The multiple genomic regions identified by GWAS for flesh color suggest that carotenoid metabolism is a complex trait governed by multiple genes acting in concert, some of them likely being *TCP* genes and other transcription factor genes. Based on the co-location of the core regulatory transcription factor from two independent color mutants, association mapping and evolutionary analysis using citrus germplasm resources, we demonstrated that TCP7 is a key transcriptional regulator that controls flesh color in citrus. TCP7 was reported to promote flowering (Li et al. 2021) and regulate endoreduplication and leaf size in *Arabidopsis thaliana* (Wang et al. 2024). During citrus evolution, Cg*TCP7* has been subjected to selection, during which a pomelo-specific allele arose. CgTCP7 can bind to the promoter of multiple carotenogenic genes, with differences in the strength of interaction and transcriptional output between the two protein variants encoded by each Cg*TCP7* allele. Multiple CgTCP transcription factors (CgTCP3, CgTCP7, and CgTCP20) and different alleles of *CgTCP7* may have different effects on the metabolic flux of the carotenoid pathway.

The red-fleshed and orange-fleshed mutants both showed higher biosynthesis of the metabolites upstream of lycopene. However, moderate up-regulation of carotenoid biosynthetic genes were detected, and the discrepancy between these moderate differences and the obvious variations observed in carotenoid contents may be attributed to the fact that the up-regulation of genes from different nodes of the pathway may synergistically strengthen the carotenoid biosynthesis. We also confirmed that the difference between fruits with red or orange flesh is due to the relative accumulation of α-carotenoid and β-carotenoid. Indeed, lycopene is the precursor for α-carotenoid and β-carotenoid biosynthesis in different branches, with the red-fleshed mutant having comparable expression of genes in both α- and β-carotenoid branch, while the orange-fleshed mutant has higher expression of genes for the β-carotenoid branch.

Similar to the typical chemically induced mutants generated in model plants, somatic mutants, with a largely identical genomic background, are an ideal system for the identification of regulatory genes related to mutant traits (Wang et al. 2021). In this study we adopted a multi-omics strategy to identify the core transcription factors regulating the mutant phenotypes. Transcriptome and transcriptional regulome (ATAC-seq in this study), can reveal the most significant and important mutational features across different molecular levels. Subsequently, defining the mutational landscape of core transcriptional regulatory genes may allow the critical mutant traits related molecular mechanism to be found. Moreover, by integrating genotypic and phenotypic data from germplasm resources, we can analyze the presence/absence, allele frequency and evolutionary selective pressures of the core regulatory genes behind the mutated traits in somatic mutants. Therefore, for crops that reproduce asexually and for which numerous somatic mutants are available, designing targeted omics experiments for meta-analysis will be a very promising approach to discover more naturally occurring desirable and previously uncharacterized alleles to promote breeding with superior and diversified quality.

## Methods

### Material preparation and sequencing

Fruit and leaf samples of white-fleshed Guanxi pomelo, red-fleshed Guanxi pomelo, and orange-fleshed Guanxi pomelo were collected at 30, 60, 90, 120 (mid-July, the color-changing period), 150, and 180 days after flowering (DAF). Each sample was collected from three different trees representing three biological replicates. At least ten fruits were collected from each tree and mixed as one biological replicate for a sample. The juice sacs were obtained, immediately frozen in liquid nitrogen, and stored in a −80°C ultra-low temperature freezer.

For the juice sacs of Guanxi pomelo wild type and its mutants at 120 days after flowering, ATAC-seq was separately performed with two biological replicates for each material (average sequencing data of 17Gb). Simultaneously, RNA was extracted separately and transcriptome sequencing (RNA-seq) was performed on the Illumina platform with three biological replicates for each material (average sequencing data of 8Gb). For the leaves of wild type and mutants of Guanxi pomelo, total DNA was extracted using a modified CTAB method, and second-generation sequencing was performed on the Illumina platform (average sequencing depth of 30 ×). HiFi sequencing was also conducted on the PacBio platform (average sequencing depth of 20 ×). For Guanxi pomelo, Hi-C libraries was constructed and sequenced on the Illumina HiSeq platform (sequencing data of 25Gb). The information of sequencing data are shown in Table S2.

### Measurement of carotenoid content of fruits

For juice sac samples of Guanxi pomelo wild type and its mutants at different developmental stages (60, 90, 120, 150, and 180 DAF), carotenoids were extracted separately, then the types and contents of carotenoids were determined by HPLC (Liu et al. 2007a, b).

### Real-time quantitative PCR analysis

Quantitative reverse transcription-PCR was performed with the SYBR-Green PCR Master Mix (YEASEN, China) using gene-specific primers on the Roche LightCycler® 480 system (Roche, China). For each sample, quantifications were conducted with three biological replicates and three technical replicates. Primer information is given in Table S8. The Actin gene was used as internal control. The qRT-PCR data were analysed using the 2-ΔΔCt.

### Genome assembly and annotation

A total of 23Gb merged HiFi reads of wild type Guanxi honey pomelo and the red-fleshed and orange-fleshed mutants were used to perform primary and phased assembly using hifiasm software (Cheng et al. 2021). Then, the preliminary contigs were assembled to super-scaffolds with HiC reads using 3D-DNA software (Dudchenko et al. 2017). The improper assemblies were manually adjusted with juicebox (URL 1), the final assemblies were performed using 3D-DNA software.

For repeat sequences annotation, simple repeats were annotated firstly by RepeatMasker software (URL 2). Then, repeat elements of viridiplantae from RepBase were annotated. The species-specific repeat library was constructed by RepeatModeler software (Flynn et al. 2020), and used to annotate species-specific repeats. Finally, above repeats annotation were combined to generate full mask results.

For gene model annotation, ab initio gene prediction, protein homolog annotation and transcriptome-based annotation were integrated. The ab initio gene prediction was performed using AUGUSTUS software (Stanke et al. 2008), SNAP software (Korf 2004) and glimmerHMM software (Majoros et al. 2004). The gene structures were annotated with published proteins of *Citrus* species by GenomeThreader software (Gremme et al. 2005). Transcriptomic data from mixed root, stem, leaf, fruit, seed, and flower was aligned to the genome using HISAT2 software (Kim et al. 2015). Then, transcriptome alignment was subjected to genome guided transcripts assembly by Trinity software (Grabherr et al. 2011) and the transcripts assembly was further refined by PASA software (Haas et al. 2003). Above gene prediction and annotation results were integrated by EVM software (Haas et al. 2008), then UTR and alternative splicing isoforms were updated by PASA software.

### Identification of genetic variation

For each of wild type Guanxi honey pomelo and the red-fleshed and orange-fleshed mutants, the HiFi reads were mapped to the merged and two haplotypes of Guanxi pomelo genome by minimap2 software (Li 2018) separately. To phase reads, the AS (alignment score) and NM (number of mismatches and gaps) alignment tags of each read between two haplotypes were compared, reads were tagged to the haplotype that aligned with higher AS and lower NM. The alignment of phased reads of white-fleshed, red-fleshed, and orange-fleshed pomelos were used to perform SNP/Indel and SV joint calling on two haplotypes by DeepVariant software (Poplin et al. 2018) and Cue software (Popic et al. 2023), respectively. Genetic variation was identified by pairwise genotype comparison. Functional effects of genetic variants were annotated using BCFtools software (Danecek et al. 2021).

### Identification of core transcription factors related to somatic mutation by combining ATAC-seq and RNA-seq data

Using the pomelo genome as the reference genome, the transcriptome sequencing data were aligned to the pomelo reference genome using TopHat software (Trapnell et al. 2012). The gene expression levels were calculated and the significantly differentially expressed genes were identified using Cufflinks software (Trapnell et al. 2012). Then, GO enrichment analyses were performed using agriGO software (Tian et al. 2017). The differential expression of the carotenoid biosynthetic pathway across red-, orange-, and white-flesh Guanxi pomelo samples was tested using the PLAGE program based on the gene expression matrix of carotenoid biosynthetic pathway genes (Tomfohr et al. 2005). To account for scale differences across genes, expression levels were standardized using Z-score normalization. Singular value decomposition (SVD) was then applied to the normalized matrix, and the first principal component was extracted as a pathway activity score for each sample. To assess the biological relevance of observed differences, Cohen’s d value was computed as a standardized measure of effect size.

The Nextera transposase adapters of ATAC-seq were removed and low-quality data was filtered out using trim_galore (URL 3). High-quality sequencing data were aligned to the pomelo reference genome using bowtie2 software (Langmead and Salzberg 2012), and the alignment results of each biological replicate of the same material were merged. High-quality reads after removing PCR duplicates and filtering of reads mapped to mitochondria and chloroplasts were obtained using SAMtools software (Li et al. 2009). Unique reads mapped to the reference genome were selected using Sambamba software (Tarasov et al. 2015). MACS2 software (Zhang et al. 2008) was used to identify transcription factor binding site peaks. The peaks were annotated using ChIPseeker software (Yu et al. 2015), the distribution of peaks on chromosomes was visualized using IGV software (Robinson et al. 2011), and motif analysis were performed using Homer software (Heinz et al. 2010). Compared to the wild type, DiffBind software (URL 4) was used to identify significantly different peaks in the red-fleshed and orange-fleshed mutants separately. The footprinting analyses of transcription factors in each material were performed, and transcription factors with significantly different footprint patterns were identified in the red-fleshed and orange-fleshed mutants compared to the wild type using HINT-ATAC in RGT software (Li et al. 2019).

By combining the differentially open chromatin regions and their binding transcription factors, transcription factors showing differential footprint patterns, transcriptional expression patterns of downstream genes, as well as carotenoid synthesis pathways and related gene information, the significantly changed "transcription factor-downstream binding gene" regulatory pathways and genes in the mutant compared to the wild type were identified.

### Identification and phylogenetic analysis of members of the TCP transcription factor family

The candidate TCP protein members were searched by the hidden Markov model (HMM) profile of TCP domain (PF03634) against protein sequences of all genes in pomelo using HMMER software (E-value < 1.0) (Potter et al. 2018). The conserved TCP domains in the candidate TCP protein sequences of pomelo were verified using the PROSITE online program (Sigrist et al. 2013), and proteins lacking TCP domains were removed.

The amino acid sequences of TCP proteins from *Arabidopsis thaliana* were downloaded from the Arabidopsis Information Resource (TAIR) database. All the TCP protein sequences from pomelo and *Arabidopsis thaliana* were aligned using CLUSTAL software (Larkin et al. 2007). A phylogenetic tree was constructed using the neighbor joining (NJ) method with a bootstrap value set to 1000 using MEGA software (Kumar et al. 2024). The phylogenetic tree was edited using the iTOL online program (Letunic and Bork 2024).

### Protein purification and electrophoretic mobility shift assay (EMSA) assays

The coding sequence encoding four candidate *TCP* was cloned into PET32a vector (His-tag), respectively. These constructs were transformed into *E. coli* BL21 (DE3), and protein expression was induced by 0.1 mM isopropyl β-D-1-thiogalactopyranoside. Protein purification was conducted using Ni NTA Beads 6FF (for His-tag protein) following the protocol by Smart Life Sciences (Changzhou, China). The primers used in the vector construction are shown in Table S8.

For EMSA assays, approximately 40-bp promoters containing TCP binding site from the *ZDS* promoter, *BCH* promoter, and *NCED2* promoter were synthesized as single-stranded oligonucleotides (5’FAM-labelled) by the Sangon Biotech (Shanghai, China). Competitive probes and mutated TCP binding site probes were unlabeled. 5’FAM-labelled EMSA assays were performed as described previously (Zhu et al. 2017) Probes information is given in Table S8.

### Transient dual-luciferase assay

For a dual luciferase reporter assay, to generate the effector vector, the coding sequences of *TCP7*^*T*^ and *TCP7*^*C*^ were amplified by PCR using genomic cDNA of white-flesh Guanxi pomelo and cloned into the effector vector (pK7WG2D) driven by *35S* promoter. The *35S:TCP7*^*T*^, *35S: TCP7*^*C*^ and empty vector (EV) were used for the effector vectors. To generate the *ZDSpro-LUC*, *BCHpro-LUC* and *NCED2pro-LUC* reporter vectors, the putative promoters (approximately 1 kb upstream of the ATG start codon of these genes) were amplified by PCR using genomic DNA of white-fleshed Guanxi pomelo as templates. The primers used in the vector construction are shown in Table S8.

The corresponding fragments were inserted into pGreen0800-LUC using Clone Express II OneStep Kit, respectively (Vazyme, China). The assigned combinations of effector and reporter were transiently co-expressed in 5-week-old tobacco (*N. benthamiana*). Luminescence signals were observed 3 days after infiltration using a living imaging apparatus (NightShade, LC985) following the instructions.

### GWAS analysis on the color phenotype of pomelo populations and evolutionary selection analysis of candidate genes

DNA sequencing data of 663 citrus accessions with an average depth of 30 × and 191 natural pomelo accessions with an average depth of 5 × were collected from published articles.

For the sequencing data of 191 natural pomelo accessions, the DNA sequencing data of each pomelo accession were aligned to the pomelo reference genome using BWA software (Li and Durbin 2009),. The SNP detection and genotype calling based on the population were conducted using Freebayes software (Garrison and Marth 2012). PLINK software (Purcell et al. 2007) was used to filter the original SNP dataset, with the screening criteria set as missing genotype rate less than 0.2, Hardy–Weinberg equilibrium test significance *P*-value less than 1e-6, and minor allele frequency greater than 0.05.

Meanwhile, 19 pomelo accessions with 50 × depth DNA sequencing were individually aligned to the pomelo reference genome for genotype detection. The information of two haplotypes in each sample was calculated using fastPHASE software (Scheet and Stephens 2006) to serve as the reference panel for imputation of missing genotype data. SHAPEIT software (Delaneau et al. 2013) was utilized to impute the missing genotype data of the pomelo population, resulting in 1,996,549 base positions for association analysis after imputation.

The SNP dataset of the pomelo population was pruned for LD using PLINK software to obtain mutually independent SNP data for population structure analysis. The PCA analysis was performed using the smartpca program in EIGENSOFT software (Price et al. 2006), and the kinship between pairs of materials was calculated using PLINK software.

Both the linear mixed model (LMM) and linear model (LR) in FaST-LMM software (Lippert et al. 2011) were employed to conduct association analysis between flesh color and SNP loci, with the flesh color phenotype divided into red and non-red types. The PCA results were used as a covariate, and the kinship K matrix was input as genetic similarity. SNP loci that met the significance threshold of *P*-value less than 0.05 and conformed to the BH test threshold (7.6) were considered associated with flesh color. Genes and their functional annotations within a 300kb region upstream and downstream of significant loci were retrieved.

For the sequencing data of 663 citrus accessions, the DNA sequencing data of each accession were aligned to the pomelo reference genome using BWA software, and genotype calling based on the population were conducted using SAMtools software. For the candidate genes and interested SNPs, the nucleotide polymorphism (π) and allele frequency (AF) were calculated using VCFtools software (Danecek et al. 2011).

## Supplementary Information

Supplementary Material 1: Fig. S1 Experimental validation of SNPs identified in red-fleshed mutant (A) and orange-fleshed mutant (B) using PCR amplification and sequencing. Fig. S2 Statistics on effects of DNA variations in red-fleshed and orange-fleshed mutants on gene structure and protein sequence. Fig. S3 Comparison of carotenoid synthesis gene sequences among different color mutants of Guanxi honey pomelo. Fig. S4 Enrichment of chromatin accessibility reads near transcription start sites (TSSs) in red-fleshed pomelo (A), orange-fleshed pomelo (B), and white-fleshed pomelo (C). Fig. S5 The significant differential footprint patterns of transcription factors in the mutants (A and B for red-fleshed and orange-fleshed mutant, respectively) and footprint patterns of some representative transcription factors enriched in the differential open regions in red-fleshed (C-D) and orange-fleshed (E-F) color mutants compared with wild type. Fig. S6 The neighbor joining (NJ) phylogenetic tree of TCPs from pomelo and Arabidopsis thaliana. Fig. S7 CDS sequence (A) and protein sequence (B) of the two alleles of CgTCP7. Fig. S8 EMSA analysis of interaction between CgTCP3 and its target promoters of ZDS and NCED2. Fig. S9 EMSA analysis of interaction between CgTCP7 and its target promoters of ZDS and BCH. Fig. S10 EMSA analysis of interaction between CgTCP20 and its target promoters of ZDS, BCH, and NCED2. Fig. S11 Gene expression patterns of CgTCP3, CgTCP7, and CgTCP20 in Guanxi honey pomelo and its color mutants in different fruit development stages (stage 1-5). Fig. S12 Verification of interaction between CgTCP7 and target carotenoid biosynthetic genes using dual-luciferase reporter assay in tobacco.

Supplementary Material 2: Table S1 Gene expression levels of carotenoid biosynthetic genes in pomelo fruits with different flesh colors during color change based on quantitative RT-PCR. Table S2 Sequencing data used in this study. RF, red-fleshed; OF, orange-fleshed; WF, white-fleshed. Table S3 Statistics of DNA variations identified in color mutants of Guanxi pomelo. Table S4 Enrichment of transcription factor motifs in differential accessible chromatin regions between red-fleshed color mutant and white-fleshed wild type. Table S5 Enrichment of transcription factor motifs in differential accessible chromatin regions between orange-fleshed color mutant and white-fleshed wild type. Table S6 Expression levels (FPKM values) of seven candidate TCP genes in Guanxi pomelo with different colors. Table S7 Prediction of transcription factor binding elements in the promoter regions of the carotenoid biosynthetic genes with differential expression levels, namely NCED2, BCH, and ZDS. Table S8 Information of primers uesed in this study.

## Data Availability

All raw sequencing data in this study have been deposited in NCBI under accession PRJNA1131555. Genome assemblies have been deposited in the NCBI under accession JBLANY000000000. The detailed accession numbers of the whole-genome sequencing data are listed in Table S2.

## Abbreviations

*TCP*: *TEOSINTE BRANCHED 1/CYCLOIDEA/PCF*
SNP: Single-nucleotide polymorphism
CNV: Copy number variation
InDel: Insertion and deletion
*GCC1*: *GARP and coiled-coil 1*
ATAC-seq: Assay for transposase-accessible chromatin with sequencing
DAF: Days after fertilization
RT-qPCR: Reverse-transcription quantitative PCR
ZDS: *ZETA-CAROTENE DESATURASE*
BCH: *BETA CAROTENOID HYDROXYLASE*
NCED2: *NINE-CIS-EPOXYCAROTENOID DIOXYGENASE 2*
LCYE: *LYCOPENE BETA-CYCLASE E*
Hi-C: High-resolution chromosome conformation capture
BUSCO: Benchmarking universal single-copy orthologs
TSS: Transcription start site
TTS: Transcription termination site
GO: Gene ontology
LUC: Luciferase
EMSA: Electrophoretic mobility shift assay
RF: Red-fleshed
OF: Orange-fleshed
WF: White-fleshed
HMM: Hidden Markov model
TAIR: The Arabidopsis Information Resource
NJ: Neighbor joining
GWAS: Genome-wide association study
