# Supplementary material for: Multi-omics analysis of somatic mutants reveals TCP7 allelically regulates multiple carotenogenic genes in citrus
Source: Mol Hortic. 2026 Feb 10;6:13. doi: 10.1186/s43897-025-00193-9 (PMC12888363; doi:10.1186/s43897-025-00193-9)
Supplement: Supplementary file 1 — Supplementary Material 1: Fig. S1 Experimental validation of SNPs identified in red-fleshed mutant (A) and orange-fleshed mutant (B) using PCR amplification and sequencing. Fig. S2 Statistics on effects of DNA variations in red-fleshed and orange-fleshed mutants on gene structure and protein sequence. Fig. S3 Comparison of carotenoid synthesis gene sequences among different color mutants of Guanxi honey pomelo. Fig. S4 Enrichment of chromatin accessibility reads near transcription start sites (TSSs) in red-fleshed pomelo (A), orange-fleshed pomelo (B), and white-fleshed pomelo (C). Fig. S5 The significant differential footprint patterns of transcription factors in the mutants (A and B for red-fleshed and orange-fleshed mutant, respectively) and footprint patterns of some representative transcription factors enriched in the differential open regions in red-fleshed (C-D) and orange-fleshed (E-F) color mutants compared with wild type. Fig. S6 The neighbor joining (NJ) phylogenetic tree of TCPs from pomelo and Arabidopsis thaliana. Fig. S7 CDS sequence (A) and protein sequence (B) of the two alleles of CgTCP7. Fig. S8 EMSA analysis of interaction between CgTCP3 and its target promoters of ZDS and NCED2. Fig. S9 EMSA analysis of interaction between CgTCP7 and its target promoters of ZDS and BCH. Fig. S10 EMSA analysis of interaction between CgTCP20 and its target promoters of ZDS, BCH, and NCED2. Fig. S11 Gene expression patterns of CgTCP3, CgTCP7, and CgTCP20 in Guanxi honey pomelo and its color mutants in different fruit development stages (stage 1-5). Fig. S12 Verification of interaction between CgTCP7 and target carotenoid biosynthetic genes using dual-luciferase reporter assay in tobacco. [file 43897_2025_193_MOESM1_ESM.docx]

**Fig. S1 Experimental validation of SNPs identified in red-fleshed mutant (A) and orange-fleshed mutant (B) using PCR amplification and sequencing.** RF, red-fleshed; OF, orange-fleshed; WF, white-fleshed. The coordinates were lifted over to HWB pomelo.

**Fig. S2 Statistics on effects of DNA variations in red-fleshed and orange-fleshed mutants on gene structure and protein sequence.** DNA variations include SNP, insertion and deletion (InDel), and structural variation (SV). RF, red-fleshed; OF, orange-fleshed; WF, white-fleshed.

**Fig. S3 Comparison of carotenoid synthesis gene sequences among different color mutants of Guanxi honey pomelo.** The HiFi sequencing data were aligned to the Guanxi honey pomelo genome, and the IGV software was used to examine whether there were DNA variations in the candidate genes and their 2kb upstream and downstream sequences among different color mutants. For each synthetic gene, the three alignment panels from top to bottom represent the white-fleshed, red-fleshed, and orange-fleshed Guanxi honey pomelo, respectively.


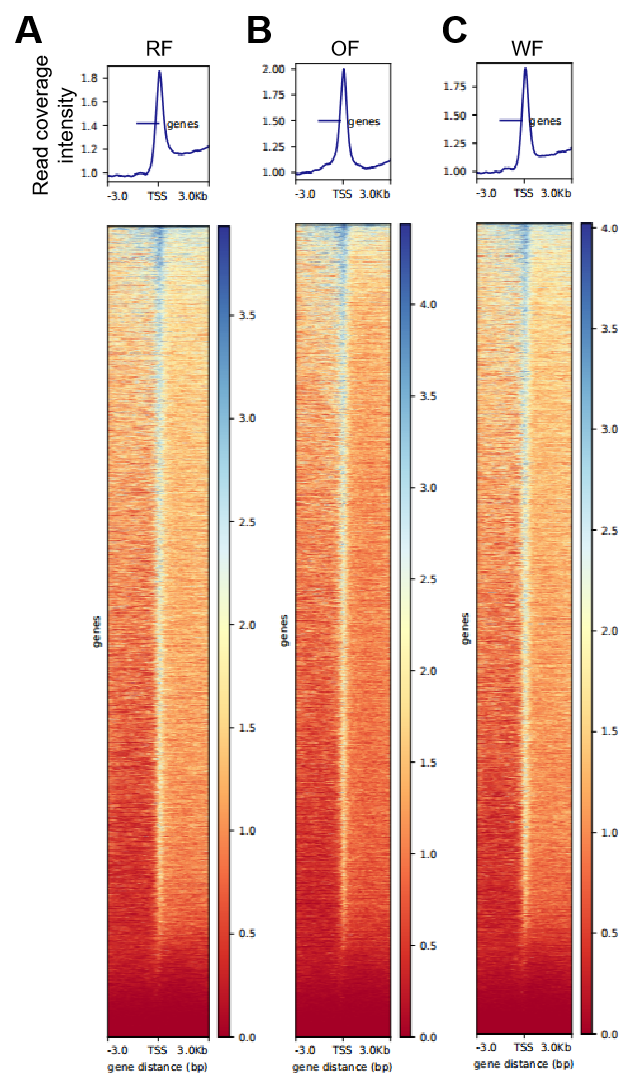


**Fig. S4 Enrichment of chromatin accessibility reads near transcription start sites (TSSs) in red-fleshed pomelo (A), orange-fleshed pomelo (B), and white-fleshed pomelo (C).**

**Fig. S5 The significant differential footprint patterns of transcription factors in the mutants (A and B for red-fleshed and orange-fleshed mutant, respectively) and footprint patterns of some representative transcription factors enriched in the differential open regions in red-fleshed (C-D) and orange-fleshed (E-F) color mutants compared with wild type.** RF, red-fleshed; YF, orange-fleshed; WF, white-fleshed.

**Fig. S6 The nighbor joining (NJ) phylogenetic tree of TCPs from pomelo and *Arabidopsis thaliana*.** The phylogenetic tree was constructed using TCP protein sequences from pomelo and *Arabidopsis thaliana*.

**Fig. S7 CDS sequence (A) and protein sequence (B) of the two alleles of *CgTCP7*.**

**Fig. S8 EMSA analysis of interaction between CgTCP3 and its target promoters of *ZDS* and *NCED2*.**

**Fig. S9 EMSA analysis of interaction between CgTCP7 and its target promoters of *ZDS* and *BCH*.**

**Fig. S10 EMSA analysis of interaction between CgTCP20 and its target promoters of *ZDS*, *BCH*, and *NCED2*.**

**Fig. S11 Gene expression patterns of *CgTCP3*, *CgTCP7*, and *CgTCP20* in Guanxi honey pomelo and its color mutants in different fruit development stages (stage 1-5).** Developmental stages 1, 2, 3, 4, and 5 correspond to 60 DAF, 90 DAF, 120 DAF, 150 DAF, and 180 DAF, respectively. W, white-fleshed; R, red-fleshed; Y, orange-fleshed.

**Fig. S12 Verification of interaction between CgTCP7 and target carotenoid biosynthetic genes using dual-luciferase reporter assay in tobacco.** (**A** and **C**) interaction between CgTCP7 and *ZDS.* Values are means ± SD (n = 8); (**B** and **D**) Interaction between CgTCP7 and *BCH*. Values are means ± SD (n = 4). The *P* values of significance of difference between CgTCP7^T^ and CgTCP7^C^ based on the t-test were indicated.
